# Can Modification with Urethane Derivatives or the Addition of an Anti-Hydrolysis Agent Influence the Hydrolytic Stability of Resin Dental Composite?

**DOI:** 10.3390/ijms24054336

**Published:** 2023-02-22

**Authors:** Agata Szczesio-Wlodarczyk, Izabela M. Barszczewska-Rybarek, Marta W. Chrószcz-Porębska, Karolina Kopacz, Jerzy Sokolowski, Kinga Bociong

**Affiliations:** 1 University Laboratory of Materials Research, Medical University of Lodz, Pomorska 251 Str., 92-213 Lodz, Poland; 2 Department of Physical Chemistry and Technology of Polymers, Silesian University of Technology, Strzody 9 Str., 44-100 Gliwice, Poland; 3 “DynamoLab” Academic Laboratory of Movement and Human Physical Performance, Medical University of Lodz, Pomorska 251 Str., 92-216 Lodz, Poland; 4 Department of Health Sciences, Medical University of Mazovia, Ludwika Rydygiera 8 Str., 01-793 Warszawa, Poland; 5 Department of General Dentistry, Medical University of Lodz, Pomorska 251 Str., 92-213 Lodz, Poland

**Keywords:** dental composites, hydrolytic stability, aging, clinical performance, urethane-dimethacrylate derivatives, anti-hydrolysis agent, CHINOX SA-1

## Abstract

Due to the questionable durability of dental restorations, there is a need to increase the lifetime of composite restoration. The present study used diethylene glycol monomethacrylate/4,4′-methylenebis(cyclohexyl isocyanate) (DEGMMA/CHMDI), diethylene glycol monomethacrylate/isophorone diisocyanate (DEGMMA/IPDI) monomers, and bis(2,6-diisopropylphenyl)carbodiimide (CHINOX SA-1) as modifiers of a polymer matrix (40 wt% urethane dimethacrylate (UDMA), 40 wt% bisphenol A ethoxylateddimethacrylate (bis-EMA), and 20 wt% triethyleneglycol dimethacrylate (TEGDMA)). Flexural strength (FS), diametral tensile strength (DTS), hardness (HV), sorption, and solubility were determined. To assess hydrolytic stability, the materials were tested before and after two aging methods (I-7500 cycles, 5 °C and 55 °C, water and 7 days, 60 °C, 0.1 M NaOH; II-5 days, 55 °C, water and 7 days, 60 °C, 0.1 M NaOH). The aging protocol resulted in no noticeable change (median values were the same as or higher than the control value) or a decrease in the DTS value from 4 to 28%, and a decrease in the FS value by 2 to 14%. The hardness values after aging were more than 60% lower than those of the controls. The used additives did not improve the initial (control) properties of the composite material. The addition of CHINOX SA-1 improved the hydrolytic stability of composites based on UDMA/bis-EMA/TEGDMA monomers, which could potentially extend the service life of the modified material. Extended studies are needed to confirm the possible use of CHINOX SA-1 as an antihydrolysis agent in dental composites.

## 1. Introduction

The literature is divided on the clinical longevity of composite dental restorations. Some sources report that premolars and molars require replacement after five or six years [1,2]. On the other hand, some researchers show that at least 60% of reconstructions made correctly with appropriate materials have a chance of surviving for more than ten years [3]. Due to the questionable durability of dental restorations, much research has focused on ways to increase the lifetime of composite restoration, particularly the polymer matrix, filler, and coupling agent.

The polymer matrix is one of the most important components of the composite. Due to its chemical structure, it is exposed to chemical reactions that can cause degradation. The new monomers synthesized for the needs of dentistry can be classified as (I) methacrylate monomers, (II) vinyl monomers, (III) click chemistry monomers, and (IV) ring-opening polymerization monomers [4]. There is no extensive research into how new polymer matrices are resistant to long-term use in the oral cavity. New monomers are characterized by certain desirable features such as low water sorption values, high values of the degree of conversion or a homogeneous structure, which may increase resistance to hydrolytic degradation.

Compared to the matrix, the filler plays a greater role in the development of the strength of the composite material. One promising trend in filler research is nanotechnology. Nanofillers are characterized by various shapes and morphology, and using them as a co-filler may have a positive effect on improving the structure and degree of filling. A certain combination of micron size fillers (or nanoclusters) with a nano size filler has been shown to exhibit the best packing, yielding very good mechanical properties and increased abrasion resistance [5,6]. A high degree of filling and homogeneity of the system increase the stability of composite materials over time [7].

The last issue related to the longevity of dental composites in the oral environment is the coupling agent. The filler compatibility in a dental composite can be improved by chemical surface modification, typically with silanes [8,9,10]. Hydrolysis can be reduced at the matrix–filler interface by increasing the hydrophobicity of the silane molecule. This can be done using molecules with an alkoxy group instead of the C=C bond, e.g., in octyltrimethoxysilane [11]. Additionally, the so-called cross-linking silanes can be used, which contain two silicon atoms each with three alkoxy groups, e.g., bis-1,2-(triethoxysilyl) ethane, or bis-1,6-(trichloroxysilyl) ethane. These compounds are able to form extensive networks that hinder the diffusion of molecules into the bulk of the material, thus increasing the hydrolytic stability of the dental composite [12].

Composite materials introduced into the market must be evaluated as biomaterials, and they are often evaluated using the ISO 4049 standard. However, such evaluation is limited and it cannot be predicted how the material will behave during long-term use in the oral environment, which due to its variable temperature and pH, friction, and various biological factors, will limit the time of the restoration [13]. Research using complex and aggressive environmental factors is very popular in other industries to determine a product’s lifetime. Hence, there is a need to evaluate the stability of dental materials in a complex operating environment when developing new materials [14,15,16,17].

**Figure 1:**
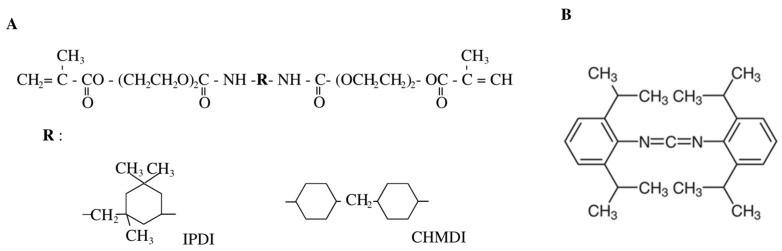
Structures of monomers (**A**) (DEGMMA/CHMDI—diethylene glycol monomethacrylate/4,4′-methylenebis(cyclohexyl isocyanate), DEGMMA/IPDI—diethylene glycol monomethacrylate/isophorone diisocyanate) and (**B**) CHINOX SA-1—bis(2,6-diisopropylphenyl)carbodiimide, used as modifiers.

The present study used diethylene glycol monomethacrylate/4,4′-methylenebis(cyclohexyl isocyanate) (DEGMMA/CHMDI) and diethylene glycol monomethacrylate/isophorone diisocyanate (DEGMMA/IPDI) monomers developed by Prof. I. Barszczewska-Rybarek [18]. These monomers are characterized by good strength properties and relatively low water sorption (Table 1). The structure of the DEGMMA/CHMDI and DEGMMA/IPDI monomers used in the study is presented in Figure 1A.

In addition, being a pilot study, bis(2,6-diisopropylphenyl)carbodiimide (CHINOX SA-1) was added to the composite as an anti-hydrolysis agent in order to improve its hydrolytic stability (Figure 1B). The industry uses such additives that can increase the stability of polymeric materials [20]. Although its chemical structure may reduce its composite biocompatibility, it seems reasonable to conduct research with the use of minor additives not yet used in dental composites.

The authors did not find information in the literature indicating the use of both urethane derivatives and an antifhydrolysis agent as modifiers of composites based on UDMA, bis-EMA, and TEGDMA monomers. In addition, studies evaluating the durability of new experimental dental composites are not a common approach; however, taking into account the methods of evaluating materials in other industries, such an assessment should be a standard procedure. The null hypothesis was that the DEGMMA/CHMDI and DEGMMA/IPDI monomers or the agent CHINOX SA-1 would not affect the properties or the hydrolytic stability of the composite assessed based on two aging protocols.

## 2. Results

The data regarding the composite modified with DEGMMA/CHMDI and DEGMMA/IPDI are presented in Table 2.

The applied aging protocols had some impact on the selected materials compared to the control group. In all samples, a significant difference was noted for the hardness values after aging.

**Figure 2:**
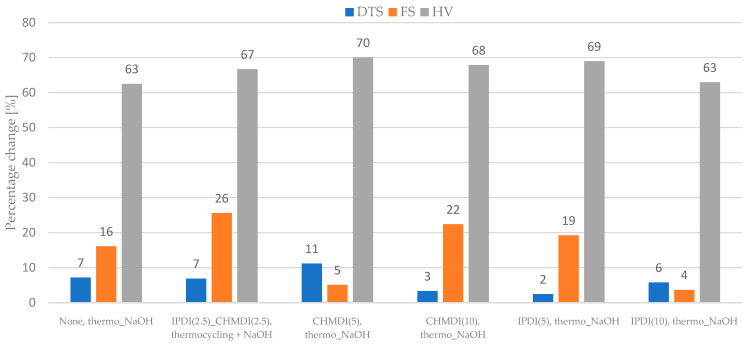
Percentage change in the flexural strength (FS), diametral tensile strength (DTS), and hardness (HV) after the thermo_NaOH aging protocol (7500 cycles, 5 °C and 55 °C, water and 7 days, 60 °C, 0.1 M NaOH) compared to control values.

**Figure 3:**
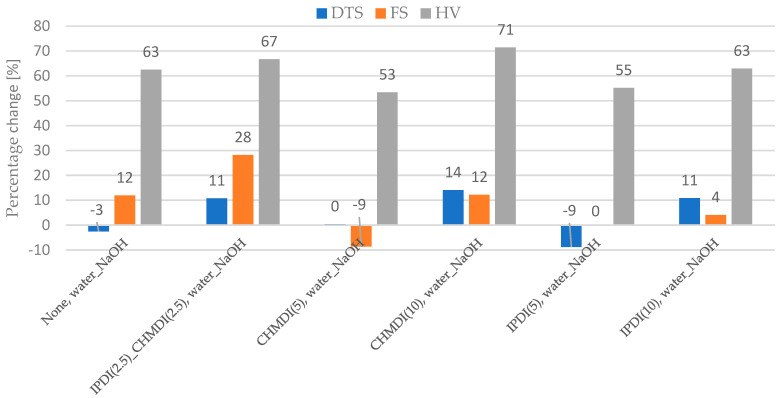
Percentage change in the flexural strength (FS), diametral tensile strength (DTS), and hardness (HV) after the water_NaOH aging protocol (5 days, 55 °C, water and 7 days, 60 °C, 0.1 M NaOH) compared to the control value. 0—no change; negative value—the selected property was higher than the control value after application of the protocol.

The percentage changes of the measured properties of composites modified with the DEGMMA/CHMDI and DEGMMA/IPDI monomers after the thermo_NaOH aging protocol and water_NaOH aging protocol are presented on Figure 2 and Figure 3, respectively.

The thermo_NaOH aging protocol (7500 cycles, 5 °C and 55 °C, water and 7 days, 60 °C, 0.1 M NaOH) yielded greater changes than the water_NaOH aging protocol (5 days, 55 °C, water and 7 days, 60 °C, 0.1 M NaOH).

The obtained results of the composite modified with the CHINOX SA-1 anti-hydrolysis agent are presented in Table 3.

After application of the aging protocols, significant changes were observed in the hardness.

**Figure 4:**
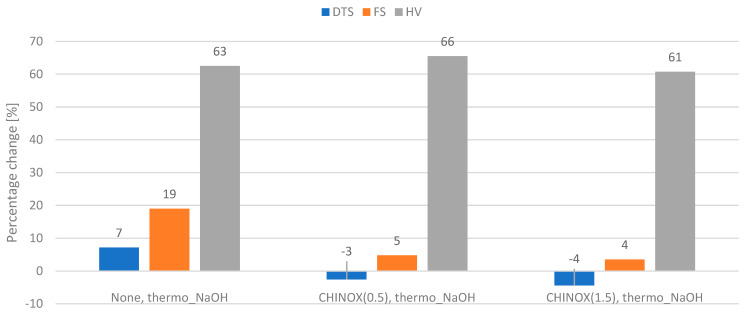
Percentage change in the flexural strength (FS), diametral tensile strength (DTS), and hardness (HV) after the thermo_NaOH aging protocol (7500 cycles, 5 °C and 55 °C, water and 7 days, 60 °C, 0.1 M NaOH) compared to controls. 0—no change; negative value—the value was higher than the control values after application of the protocol.

**Figure 5:**
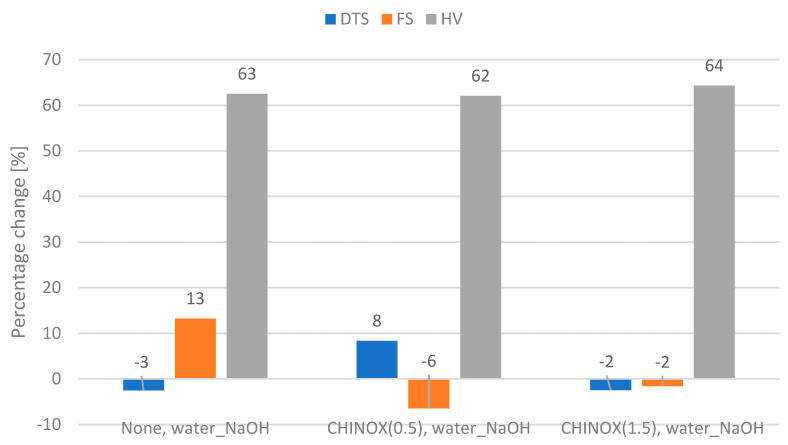
Percentage change in the flexural strength (FS), diametral tensile strength (DTS), and hardness (HV) after the water_NaOH aging protocol (5 days, 55 °C, water and 7 days, 60 °C, 0.1 M NaOH) compared to controls. 0—no change; negative value—the value was higher than the control values after application of the protocol.

The percentage changes in the measured properties of the composites modified with CHINOX SA 1 after the thermo_NaOHaging protocol and water_NaOH aging protocol are presented on Figure 4 and Figure 5, respectively.

The CHINOX SA-1-modified materials yielded smaller percentage changes compared to the non-modified control material.

**Figure A1:**
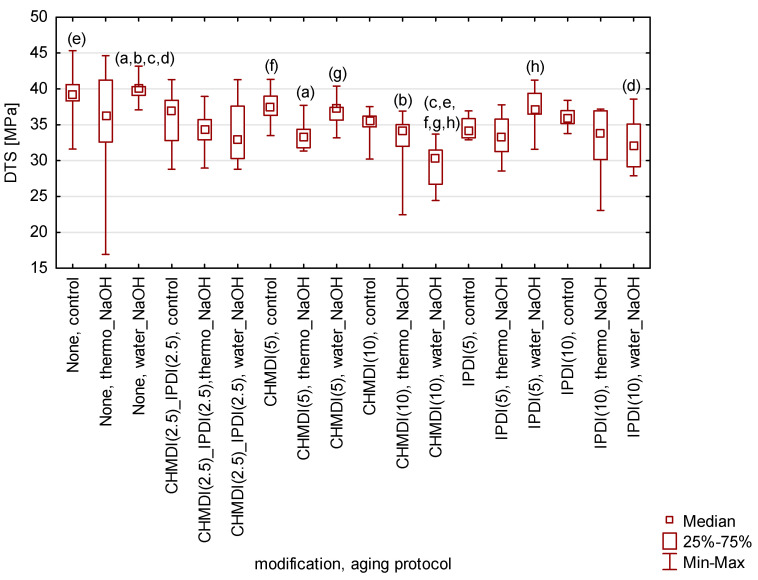
Box-and-whisker plot of the diametral tensile strength (DTS) of the tested materials. Statistically significant differences were detected between the following: (a,b,c,d) None, water_NaOH vs. (a) CHMDI(5), thermo_NaOH (*p* = 0.0261), (b) CHMDI(10), thermo_NaOH (*p* = 0.0272), (c) CHMDI(10), water_NaOH (*p* = 0.00002), (d) IPDI(10), water_NaOH (*p* = 0.0087); (e,f,g,h) CHMDI(10), water_NaOH vs. (e) None, control (*p* = 0.0005), (f) CHMDI(5), control (*p* = 0.0082), (g) CHMDI(5), water_NaOH (*p* = 0.0421), (h) IPDI(5), water_NaOH (*p* = 0.0095).

**Figure A2:**
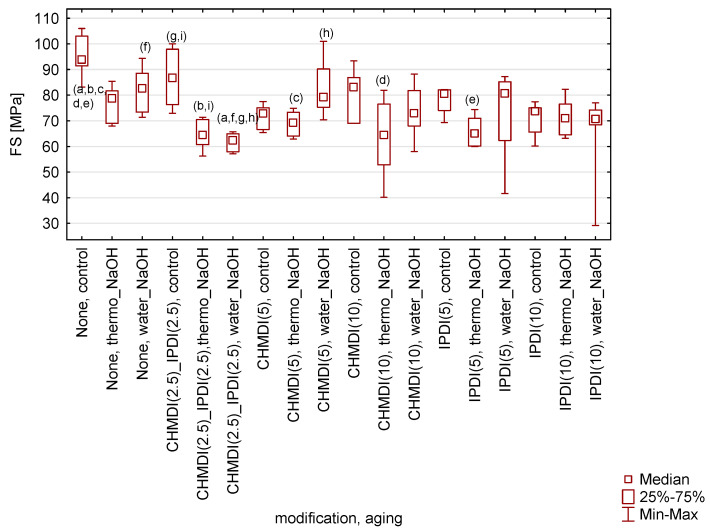
Box-and-whisker plot of the flexural strength (FS) of the tested materials. Statistically significant differences were detected between the following: (a,b,c,d,e) None, control vs. (a) CHMDI(2.5)_IPDI(2.5), water_NaOH (*p* = 0.00004), (b) CHMDI(2.5)_IPDI(2.5), thermo_NaOH (*p* = 0.0005), (c) CHMDI(5), thermo_NaOH (*p* = 0.0151), (d) CHMDI(10), thermo_NaOH (*p* = 0.0103), (e) IPDI(5), thermo_NaOH (*p* = 0.0034); (a,f,g,h) CHMDI(2.5)_IPDI(2.5), water_NaOH vs. (f) None, water_NaOH (*p* = 0.0165), (g) CHMDI(2.5)_IPDI(2.5), control (*p* = 0.0051), (h) CHMDI(5), water_NaOH (*p* = 0.0254); (i) CHMDI(2.5)_IPDI(2.5), control vs. (i) CHMDI(2.5)_IPDI(2.5), thermo_NaOH (*p* = 0.0459).

**Figure A3:**
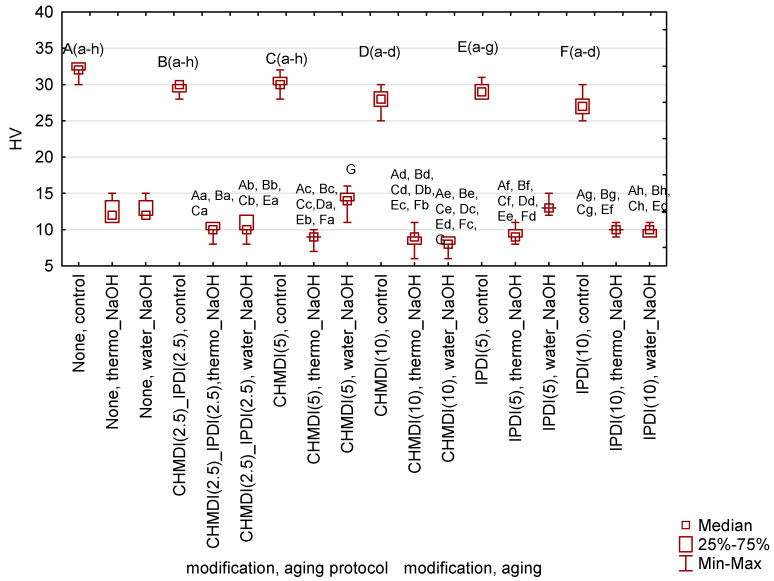
Box-and-whisker plot of the Vickers hardness (HV) of the tested materials. Statistically significant differences were detected between the following: (A(a–h)) None, control vs. (Aa) CHMDI(2.5)_IPDI(2.5), water_NaOH (*p* = 0.0005), (Ab) CHMDI(2.5)_IPDI(2.5), thermo_NaOH (*p* = 0.0004), (Ac) CHMDI(5), thermo_NaOH (*p* = 0.0000), (Ad) CHMDI(10), thermo_NaOH (*p* = 0.0000), (Ae) CHMDI(10), water_NaOH (*p* = 0.0000), (Af) IPDI(5), thermo_NaOH(0.0000), (Ag) IPDI(10), thermo_NaOH (*p* = 0.0001), (Ah) IPDI(10), water_NaOH (*p* = 0.0001); (B(a–h)) CHMDI(2.5)_IPDI(2.5), control vs. (Ba) CHMDI(2.5)_IPDI(2.5), water_NaOH (*p* = 0.0298), (Bb) CHMDI(2.5)_IPDI(2.5), thermo_NaOH (*p* = 0.0212), (Bc) CHMDI(5), thermo_NaOH (*p* = 0.0001), (Bd) CHMDI(10), thermo_NaOH (*p* = 0.0001), (Be) CHMDI(10), water_NaOH (*p* = 0.0000), (Bf) IPDI(5), thermo_NaOH(0.0007), (Bg) IPDI(10), thermo_NaOH (*p* = 0.005366), (Bh) IPDI(10), water_NaOH (*p* = 0.0049); (C(a-h)) CHMDI(5), control vs. (Ca) CHMDI(2.5)_IPDI(2.5), water_NaOH (*p* = 0.0080), (Cb) CHMDI(2.5)_IPDI(2.5), thermo_NaOH (*p* = 0.0055), (Cc) CHMDI(5), thermo_NaOH (*p* = 0.0000), (Cd) CHMDI(10), thermo_NaOH (*p* = 0.0000), (Ce) CHMDI(10), water_NaOH (*p* = 0.0000), (Cf) IPDI(5), thermo_NaOH(0.0002), (Cg) IPDI(10), thermo_NaOH (*p* = 0.0013), (Ch) IPDI(10), water_NaOH (*p* = 0.0011); (D(a–d)) CHMDI(10), control vs. (Da) CHMDI(5), thermo_NaOH (*p* = 0.0022); (Db) CHMDI(10), thermo_NaOH (*p* = 0.0016), (Dc) CHMDI(10), water_NaOH (*p* = 0.0001), (Dd) IPDI(5), thermo_NaOH (*p* = 0.0106); (E(a–g)) IPDI(5), control vs. (Ea) CHMDI(2.5)_IPDI(2.5), thermo_NaOH (*p* = 0.0401), (Eb) CHMDI(5), thermo_NaOH (*p* = 0.0003), (Ec) CHMDI(10), thermo_NaOH (*p* = 0.0002), (Ed) CHMDI(10), water_NaOH (*p* = 0.0000), (Ee) IPDI(5), thermo_NaOH(0.0016), (Ef) IPDI(10), thermo_NaOH (*p* = 0.0107), (Eg) IPDI(10), water_NaOH (*p* = 0.0097); (F(a–d)) IPDI(10), control vs. (Fa) CHMDI(5), thermo_NaOH (*p* = 0.0022); (Fb) CHMDI(10), thermo_NaOH (*p* = 0.0016), (Fc) CHMDI(10), water_NaOH (*p* = 0.0001), (Fd) IPDI(5), thermo_NaOH (*p* = 0.0106); (G) CHMDI(5), water_NaOH vs. (G) CHMDI(10), water_NaOH (*p* = 0.0289).

**Figure A4:**
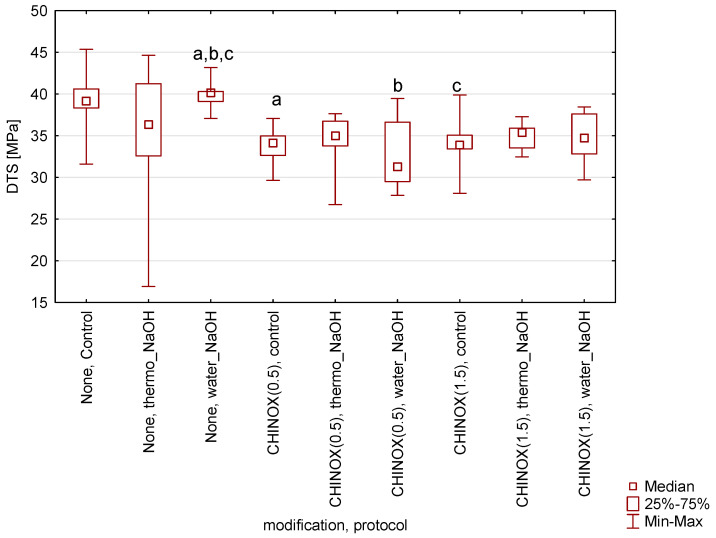
Box-and-whisker plot of the diametral tensile strength (DTS) of the tested materials. Statistically significant differences were detected between the following: (a,b,c) None, water_NaOH vs. (a) CHINOX(0.5), control (*p* = 0.0440); (b) CHINOX(0.5), water_NaOH (*p* = 0.0170), (c) CHINOX(1.5), control (*p* = 0.0340).

**Figure A5:**
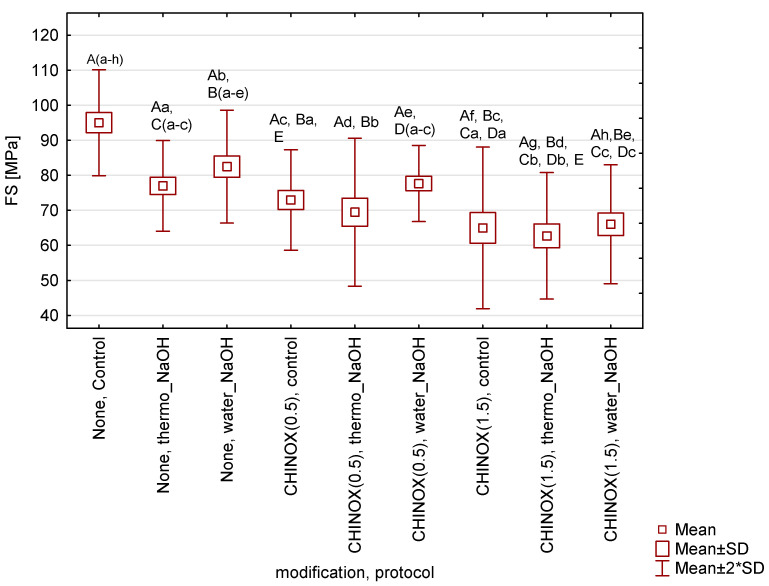
Box-and-whisker plot of the flexural strength (FS) of the tested materials. Statistically significant differences were detected between the following: A(a–h)) None, control vs. (Aa) None, thermo_NaOH (*p* = 0.0002), (Ab) None, water_NaOH (*p* = 0.0075),(Ac) CHINOX(0.5), control (*p* = 0.0000), (Ad) CHINOX(0.5), thermo_NaOH (*p* = 0.0000), (Ae) CHINOX(0.5), water_NaOH (*p* = 0.0003), (Af) CHINOX(1.5), control (*p* = 0.0000), (Ag) CHINOX(1.5), thermo_NaOH (*p* = 0.0000), (Ah) CHINOX(1.5), water_NaOH (*p* = 0.0000); B(a–e) None, water_NaOH vs. (Ba) CHINOX(0.5), control (*p* = 0.0389), (Bb) CHINOX(0.5), thermo_NaOH (*p* = 0.0057), (Bc) CHINOX(1.5), control (*p* = 0.0003), (Bd) CHINOX(1.5), thermo_NaOH (*p* = 0.0001), (Be) CHINOX(1.5), water_NaOH (*p* = 0.0006); C(a–c) None, thermo_NaOHvs. (Ca) CHINOX(1.5), control (*p* = 0.0104), (Cb) CHINOX(1.5), thermo_NaOH (*p* = 0.0026); (Cc) CHINOX(1.5), water_NaOH (*p* = 0.0187); D(a–c) CHINOX(0.5), water_NaOH vs. (Da) CHINOX(1.5), control (*p* = 0.0070), (Cb) CHINOX(1.5), thermo_NaOH (*p* = 0.0017); (Cc) CHINOX(1.5), water_NaOH (*p* = 0.0128); (E) CHINOX(0.5), control vs. (E) CHINOX(1.5), thermo_NaOH (*p* = 0.0272).

**Figure A6:**
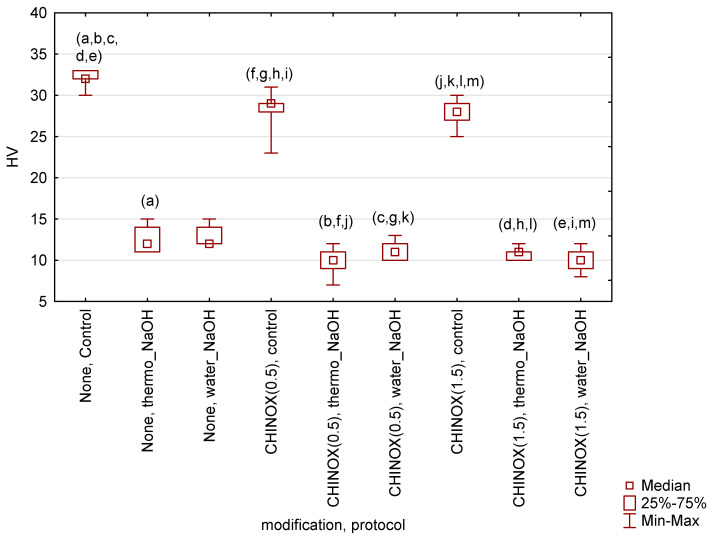
Box-and-whisker plot of the Vickers hardness (HV) of the tested materials. Statistically significant differences were detected between the following: (a,b,c,d,e) None, Control vs.(a) None, thermo_NaOH ( *p* = 0.0237), (b) CHINOX(0.5), thermo_NaOH (*p* = 0.0000), (c) CHINOX(0.5), water_NaOH (*p* = 0.0001), (d) CHINOX(1.5), thermo_NaOH (*p* = 0.0000), (e) CHINOX(1.5), water_NaOH (*p* = 0.0000); (f,g,h,i) CHINOX(0.5), control vs. (f) CHINOX(0.5), thermo_NaOH(*p* = 0.0007), (g) CHINOX(0.5), water_NaOH (*p* = 0.0143), (h) CHINOX(1.5), thermo_NaOH(*p* = 0.0059), (i) CHINOX(1.5), water_NaOH (*p* = 0.0006); (j,k,l,m) CHINOX(1.5), control vs. (j) CHINOX(0.5), thermo_NaOH (*p* = 0.0018), (k) CHINOX(0.5), water_NaOH (*p* = 0.0335), (l) CHINOX(1.5), thermo_NaOH (*p* = 0.0146), (m) CHINOX(1.5), water_NaOH (*p* = 0.0013).

Box-and-whisker plots of the collected results and exact *p*-values are provided in Appendix A (Figure A1, Figure A2, Figure A3, Figure A4, Figure A5 and Figure A6).

The sorption and solubility of the tested materials are presented in Table 4.

The applied modifications slightly increased the sorption value. The solubility of the tested materials increased.

## 3. Discussion

There is a pressing need to identify a composite with increased resistance to hydrolytic degradation and hence a longer lifetime [21,22]. It should be borne in mind that the oral environment has a significant impact on composite durability. As such, it is crucial that materials are evaluated under accelerated aging conditions with increased environmental factors.

Our findings indicate that the selected modification did indeed influence the properties of the control (base) material, thus rejecting the null hypothesis. However, the use of the DEGMMA/CHMDI, DEGMMA/IPDI monomers and the addition of CHINOX SA-1 did not improve the initial (control) strength properties. The initial values of the tested properties were lower than those of the base material, but not all differences were statistically significant.

The properties of a composite material are influenced by its composition. Considering that the materials have the same filler, the observed differences in the properties will depend on the composition of the polymer matrix [23,24]. The tested materials, which consisted of a basic polymer matrix (40 wt% of UDMA, 40 wt% of Bis-EMA, 20 wt% of TEGDMA), was modified with two monomers (DEGMMA/CHMDI, DEGMMA/IPD) in different weight percentages. As the used monomers had two oxyethylene units (DEGMMA), the molecule showed limited flexibility. Additionally, the monomers contained cycloaliphatic diisocyanates: CHMDI or IPDI, which differed in their structure symmetry. The monomer with IPDI is more elastic than the one with CHMDI, which may result in higher DC and modulus [18]. In our research, a small addition (max 10 wt%) of the cycloaliphatic urethane-dimethacrylate derivatives did not improve the strength properties. However, the TEGDMA, Bis-EMA, or UDMA homopolymers demonstrated lower flexural strength and flexural modulus than the resins used as modification (Table 1). Even so, it should be taken into account that the properties of the composite result from complex relationships and interactions between individual components that cannot be predicted under the current state of science. It is most likely that the addition of further substances could hinder the movement of macromolecules during polymerization due to their chemical structure (Figure 1A) [18]. Composite materials modified with CHMDI or IPDI monomers could achieve lower DC values resulting in lower FS and DTS values.

In addition, the modification with the anti-hydrolysis agent did not improve the control values. CHINOX SA-1 includes two phenyl groups connected to each other by a short carbodiimide group (Figure 1B). This stiff structure, similar to bis-GMA, may prevent the free movement of macromolecules during polymerization, resulting in a lower degree of conversion by the composite [25,26]. The lower DC can explain the decreased FS, DTS, and HV values and the increased sorption of modified composites with CHINOX SA-1.

Although dental materials placed on the market must meet certain requirements, such as biocompatibility, the relevant ISO and ADA standards include no tests for assessing the stability of materials in the oral cavity. Water sorption can be used to assess the behavior of materials in a water environment. The ISO 4049 standard specifies that the sorption of the composite material cannot be higher than 40 µg/mm^−3^ at a solubility of 7.5 µg/mm^−3^ [27]. For the tested modified monomers, the sorption was relatively low (Table 1). Regarding the DEGMMA/CHMDI monomer, the low sorption values are due to the presence of symmetrical cycloaliphatic moieties, which causes a reduction in the space between the polymer chains. In contrast, the asymmetric core in IPDI can create more free space in the polymer network for water to enter, resulting in higher sorption. In the study, composites modified with selected urethane derivatives showed similar sorption values as the control materials. Minor amounts of selected monomers were added (max. 10 wt%); therefore, the effect on sorption was small. The sorption values increased noticeably in comparison with the control when CHINOX SA-1 added, but these values were still at an acceptable level. There was also no difference between the addition of 0.5 and 1.5%. The solubility of the tested materials compared to the control material increased. The observed changes may also be related to the structure of the used modifiers, which could have resulted in a lower degree of the conversion values. Materials with lower DC showed higher sorption and solubility values [28].

Of the two tested aging protocols, the thermocycler approach (7500 cycles, 5 °C and 55 °C, water and 7 days, 60 °C, 0.1 M NaOH) reduced the value of the tested properties more effectively (Figure 2, Figure 3, Figure 4 and Figure 5) than water (5 days, 55 ° C, water and 7 days, 60 °C, 0.1 M NaOH). It has been shown that thermocycles affect the degradation of the polymer matrix as well as the stability of the matrix–filler interface [29,30]. Boussès et al. reported slow degradation of at the filler–matrix interface for the first 5000 thermocycles, while significant changes were noted after 10,000 thermocycles [31]. From a chemical point of view, composite samples take up water during aging, resulting in hydrolysis of the polymer matrix and interface. Firstly, the matrix protects the interface from degradation until the polymer structure is saturated with water. Once water reaches the interphase, the siloxane bonds are exposed to hydrolysis. Unfortunately, this type of bond, which results from filler silanization, is not resistant to hydrolytic degradation [10,32]. Thermocycling causes successive contractions and expansions of the material due to temperature variations. In addition, due to different thermal expansion coefficients, local overstress is generated at the interface. This may cause the occurrence of micro-cracks and interface damage, leading to a greater decrease in strength [31]. In the present study, the effect of the thermocycler was enhanced by NaOH; this causes accelerated degradation of dental polymer materials due to a high amount of hydroxyl ions, which are responsible for the hydrolysis of the bonds present in the polymer matrix, the coupling agent or the interphase [33,34,35]. It is worth mentioning that the phenomenon of degradation of dental materials does not occur only under the influence of substances and physical conditions (aqueous environment, temperature, pH, chewing forces, friction) occurring in the oral cavity. Aging of materials also develops under the influence of biological factors—salivary enzymes and bacterial activity. It has been shown that these factors also cause the hydrolysis of chemical bonds found in composite materials [36]. However, enzyme-based analyses are costly and more demanding than the proposed aging protocols.

Research has shown that the top layer is the most susceptible to aging and degradation. For most of the studied groups, the hardness values after aging were more than 60% lower than those of the controls. The aging medium reduces hardness by affecting the matrix and weakens the siloxane bonds in the silane coupling agent [37]. The alkalinity provides a large amount of hydroxyl ions, which are responsible for hydrolysis [38]. The microstructure of the dental composite changes after chemical or thermal aging. Plucking out, fractures of filler particles, and degradation–delamination of the matrix were observed [14,34,39,40]. The aging protocol resulted in no noticeable change (median values were the same as or higher than those of the control value) or a decrease in the DTS value from 4 to 28% and a decrease in the FS value by 2 to 14% (Figure 2, Figure 3, Figure 4 and Figure 5). Considering the lower percentage changes in strength, it appears that the degradation effect is more superficial and does not propagate into the bulk of the material. However it should be underlined that in the oral environment, the top aged layer will be successively lost due to the continuous friction applied by chewing forces, allowing the restoration to be continually eroded [41].

No relationship was found between the percentage composition of the polymer matrix and the hydrolytic resistance, identified by changes in selected properties. Smaller changes in DTS and FS were observed in the CHMDI(5) and the IPDI(5) and (10) groups; these values were very close to those of the control. No significant improvement in stability against hydrolytic degradation was noted for most composites, which was probably more dependent on the network structure of the materials and the complex interaction between the used monomers. The UDMA monomer and its cycloaliphatic derivatives (DEGMMA/IPDI and DEGMMA/CHMDI) have urethane bonds, which are prone to form strong hydrogen bonds. These bonds acts as physical crosslinks in the resulting polymer network. The enrichment of the studied system with DEGMMA/IPDI and DEGMMA/CHMDI may cause increase in the physical crosslinked density. Hence, monomers of the UDMA/bis-EMA/TEGDMA matrix could form less homogeneous structures, which would influence the changes in the tested properties after aging. Some inhomogeneity in materials may create a spot where a stress accumulates, resulting in microcracks and treatment failure. It should be remembered that any imperfection in the material can influence its durability.

**Figure 6:**
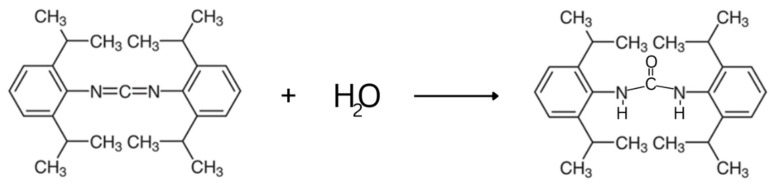
Reaction of bis(2,6-iisopropylphenyl)carbodiimide (CHINOX SA-1) with water.

The addition of CHINOX SA-1 improved the hydrolytic stability of the tested materials. The percentage changes in the DTS and FS values following aging were limited, even at low CHINOX SA-1 concentrations (0.5%). Unfortunately, no data regarding this concentration in dental materials could be found in previous studies; however, research conducted, for example, with poly (lactic acid) has shown that carbodiimide compounds increase the resistance to hydrolytic degradation due to the water reacting with anti-hydrolysis compounds, producing urea derivatives [42,43]. It is most likely that the reaction of the CHINOX SA-1 anti-hydrolysis agent and water molecule proceeds according to the equation presented in Figure 6.

As small additions have been found to be effective in increasing hydrolytic stability, it is likely that the cellular response will not be impaired. However, future studies are still needed to determine the biocompatibility of such composites.

## 4. Materials and Methods

The basic material used in this work was a polymer matrix consisting of 40 wt% UDMA, 40 wt%, bis-EMA, and 20 wt% TEGDMA. A resin matrix was prepared according to the weight percentage of the selected monomers. UDMA, TEGDMA, and bis-EMA were delivered by Esstech Inc. (Essington, PA, USA). Such a resin matrix was additionally modified with a specific amount of selected urethane monomers or anti-hydrolysis agent (Table 5). Each mixture contained camphorquinone (<1 wt%) and N, N-dimethylaminoethyl methacrylate. Monomers were synthesized, and their structure was confirmed as described previously [18,44]. CHINOX SA-1 was delivered by TCI Chemicals (Fukaya, Japan).

After modification, 45 wt% filler was added to each of the prepared polymer matrices using a mortar. The filler was silica (Arsil, Zakłady Chemiczne “RUDNIKI” S.A., Rudniki, Poland) silanized with γ-Methacryloxypropyltrimethoxy silane (Unisil Sp. Z o. O., Tarnów, Poland).

Two different ageing protocols were used (Table 6) to evaluate the hydrolytic stability of the tested materials. The flexural strength, diametral tensile strength, and hardness were determined with and without the influence of the aging protocols. The protocols were selected on the basis of previous research [14]. Briefly, hydrolytic degradation was accelerated by NaOH solution. Thermocycles and increased temperature affect sorption and dissolution. The combination of thermal and chemical factors better mimic the prolonged influence of the oral environment on restoration. Accelerated aging with more aggressive or greater amounts of factors can be used to evaluate the lifetime performance of dental composite in vivo [14,45]. It can be assumed that the proposed aging protocols will simulate several years in the oral environment; however, such a prediction is very complex and difficult to make.

The NaOH solution was prepared in a volumetric flask. The NaOH (Avantor Performance Materials, Gliwice, Poland) was measured on an analytical balance (Radwag XA 82/220/X, Puszczykowo, Poland). The samples were placed in plastic dishes in a DZ-2BCII Vacuum Drying Oven (ChemLand, Stargard Szczecinski, Poland) for a period (Table 6) or were subjected to 7500 thermocycles (water, 20 s dwell time, 5 and 55 °C) using a THE 1200 thermocycler (SD Mechatronic, Feldkirchen-Westerham, Germany).

Flexural strength (FS) was determined based on ISO 4049:2019. Seven measurements were made for each study group, using rectangular samples (25 mm long, 2 mm wide, 2 mm thick). Nine cylindrical samples (diameter 6 mm and thickness 3 mm) for each study group were used to establish the diametral tensile strength (DTS). The FS and DTS tests were performed using a Z020 universal testing machine (Zwick–Roell, Ulm, Germany). The traverse speed was 2 mm/min in the DTS test and 1 mm/min in FS. Nine measurements of Vickers hardness (1000 g applied load, 10 s penetration time) were carried out for each study group. The Zwick ZHV2–m hardness tester (Zwick–Roell, Ulm, Germany) was used in this study. In addition, water sorption and the solubility of the tested materials were evaluated, based on ISO standard (4049:2019 Dentistry—Polymer-based restorative materials). Five cylindrical samples (15 mm in diameter, 1 mm in width) were prepared for each composite.

Water sorption (W_sp_) and solubility (W_sl_) were calculated for each specimen using the following equations:

(1) $$W_{\mathrm{sp}}=\frac{m_{2}-m_{1}}{V}\cdot 100\%$$

(2) $$W_{\mathrm{sp}}=\frac{m_{1}-m_{3}}{V}\cdot 100\%$$

where m_1_ is the conditioned mass of the specimen, m_2_ is the mass of the specimen after immersion in water, m_3_ is the reconditioned mass of the specimen, and V is the specimen volume.

The Shapiro–Wilk test was used to assess the normality of the distribution of the data. Based on the results, either the Kruskal–Wallis test with multiple comparisons of mean ranks or one-way ANOVA was applied, followed by Tukey’s post hoc test. The accepted level of significance was α = 0.05. Data with a normal distribution and homogeneity of variance are presented as mean values with standard deviation (SD), while those without are presented as median values with the interquartile range (IQR). All analyses were performed using Statistica version 13 software (StatSoft, Kraków, Poland).

## 5. Conclusions

These studies examined one composite, albeit as five variants with DEGMMA/CHMDI, DEGMMA/IPDI monomers, and two modifications with the CHINOX SA-1 anti-hydrolysis agent. Further research is needed with a wider group of materials and more testing methods. Nevertheless, our findings indicate the following:

- The additives (DEGMMA/CHMDI, DEGMMA/IPDI, CHINOX SA-1) did not improve the initial (control) properties of the composite material.
- No relationship was found between the percentage composition of the polymer matrix and hydrolytic stability, tested by changes in selected properties after aging.
- The addition of CHINOX SA-1 improved the hydrolytic stability of the composites based on the UDMA/bis-EMA/TEGDMA monomers.
- In all materials, the hardness dropped dramatically after the aging protocols. This may prove that the degradation of materials takes place mainly in the top layer.

The standardization of the aging protocol for dental materials is a separate project (funded by National Science Centre, Poland grant number: UMO-2020/37/N/ST5/00191, 2021).

## Figures and Tables

**Table 1:** The properties of the used monomers: molecular weight (MW), flexural strength (FS), flexural modulus (E), water sorption (WS), degree of conversion (DC).

Monomer	MW [g/mol]	FS [MPa]	E [GPa]	WS [µg/mm^3^]	DC [%]
UDMA	470	134 ^a^	1.8 ^a^	42.3 ^a^	72 ^a^
Bis-EMA	540	87 ^a^	1.1 ^a^	21.3 ^a^	76 ^a^
TEGDMA	286	99 ^a^	1.7 ^a^	28.8 ^a^	83 ^a^
DEGMMA/CHMDI	611	139 ^b^	3.4 ^b^	18.5 ^b^	41 ^b^
DEGMMA/IPDI	571	141 ^b^	2.8 ^b^	29.9 ^b^	66 ^b^

a—taken from [19]; b—taken from [18]. UDMA—urethane dimethacrylate, bis-EMA—bisphenol A ethoxylateddimethacrylate, TEGDMA—triethyleneglycol dimethacrylate, CHINOX SA-1—bis(2,6-diisopropylphenyl)carbodiimide, DEGMMA/CHMDI—diethylene glycol monomethacrylate/4,4′-methylenebis(cyclohexyl isocyanate), DEGMMA/IPDI—diethylene glycol monomethacrylate/isophorone diisocyanate.

**Table 2:** The results of the flexural strength (FS), diametral tensile strength (DTS), and hardness (HV) of the tested materials modified with DEGMMA/CHMDI and DEGMMA/IPDI after selected aging protocols. The results with the same assigned letter or uppercase and lowercase letters are significantly different (*p* ≤ 0.05). Median values are presented with the interquartile range (IQR).

	DTS [MPa]	(IQR)	FS [MPa]	(IQR)	HV	(IQR)
Number of Samples in the Study Group	*n* = 9		*n* = 7		*n* = 9	
None, Control	39.14 ^e^	2.29	93.8 ^a,b,c,d,e^	11.6	32 ^A(a–h)^	1
None, thermo_NaOH	36.33	8.64	78.7	12.7	13	2
None, water_NaOH	40.13 ^a,b,c,d^	1.20	82.6 ^f^	15.1	13	1
IPDI(2.5)_CHMDI(2.5), control	36.90	5.60	86.7 ^g,i^	21.6	29 ^B(a–h)^	1
IPDI(2.5)_CHMDI(2.5), thermo_NaOH	34.36	2.81	64.5 ^b,i^	9.8	10 ^Ab,Bb,Cb,Ea^	1
IPDI(2.5)_CHMDI(2.5), water_NaOH	32.94	7.29	62.3 ^a,f,g,h^	7.0	10 ^Aa,Ba,Ca^	1
CHMDI(5), control	37.42 ^f^	2.66	72.9	8.4	30 ^C(a–h)^	1
CHMDI(5), thermo_NaOH	33.23 ^a^	2.56	69.2 ^c^	9.3	9 ^Ac,Bc,Cc,Da,^ ^Eb,Fa^	1
CHMDI(5), water_NaOH	37.28 ^g^	1.76	79.2 ^h^	14.9	14 ^G^	1
CHMDI(10), control	35.35	1.79	83.1	17.9	28 ^D(a-d)^	1
CHMDI(10), thermo_NaOH	34.18 ^b^	3.01	64.5 ^d^	23.7	9 ^Ad,Bd,Cd,Db,^ ^Ec,Fb^	1
CHMDI(10), water_NaOH	30.38 ^c,e,f,g,h^	4.77	73.0	13.8	8 ^Ae,Be,Ce,Dc,^ ^Ed,Fc,G^	1
IPDI(5), control	34.17	2.68	80.6	8.1	29 ^E(a–g)^	1
IPDI(5), thermo_NaOH	33.34	4.52	65.1 ^e^	10.9	9 ^Af,Bf,Cf,Dd,Ee, Fd^	1
IPDI(5), water_NaOH	37.18 ^h^	2.89	80.7	22.9	13	1
IPDI(10), control	35.99	1.80	73.7	14.9	27 ^F(a–d)^	2
IPDI(10), thermo_NaOH	33.90	6.80	71.0	12.0	10 ^Ag,Bg,Cg,Ef^	1
IPDI(10), water_NaOH	32.10 ^d^	5.91	70.7	5.8	10 ^Ah,Bh,Ch,Eg^	1

**Table 3:** The results of the flexural strength (FS), diametral tensile strength (DTS), and hardness (HV) for tested materials modified with CHINOX SA-1 after selected aging protocols. The results with the same assigned letter or uppercase and lowercase letters are significantly different. FS is presented as the mean with standard deviation (SD), while DTS and HV are presented as the median with the interquartile range (IQR).

	DTS [MPa]	IQR	FS [MPa]	SD	HV	IQR
Number of Samples in the Study Group	*n* = 9		*n* = 7		*n* = 9	
None, Control	39.14	2.29	95.0 ^A(a–h)^	7.6	32 ^a,b,c,d,e^	1
None, thermo_NaOH	36.33	8.64	77.0 ^Aa,C(a–c)^	6.5	12 ^a^	3
None, water_NaOH	40.13 ^a,b,c^	1.20	82.5 ^Ab,B(a–e)^	8.1	12	2
CHINOX(0.5), control	34.13 ^a^	2.33	73.0 ^Ac,Ba,E^	7.2	29 ^f,g,h,i^	1
CHINOX(0.5), thermo_NaOH	35.00	2.98	69.5 ^Ad,Bb^	10.6	10 ^b,f,j^	2
CHINOX(0.5), water_NaOH	31.28 ^b^	7.13	77.7 ^Ae,D(a–c)^	5.4	11 ^c,g,k^	2
CHINOX(1.5), control	33.89 ^c^	1.64	65.0 ^Af,Bc,Ca,Da^	11.5	28 ^j,k,l,m^	2
CHINOX(1.5), thermo_NaOH	35.38	2.37	62.7 ^Ag,Bd,Cb,Db,E^	9.0	11 ^d,h,l^	1
CHINOX(1.5), water_NaOH	34.71	4.81	66.0 ^Ah,Be,Cc,Dc^	8.5	10 ^e,i,m^	2

**Table 4:** The results of sorption and solubility. The results are shown as the mean with standard deviation (SD).

		Control	CHMDI(2.5)_IPDI(2.5)	CHMDI(5)	CHMDI(10)	IPDI(5)	IPDI(10)	CHINOX(0.5)	CHINOX(1.5)
Sorption	n = 5	25.42 (0.53)	28.96 (0.40)	26.55 (1.07)	32.40 (1.56)	26.81 (0.60)	32.64 (2.41)	32.97 (1.53)	32.91 (0.73)
Solubility	n = 5	0.70 (0.20)	0.68 (0.15)	1.05 (0.28)	1.46 (0.42)	1.31 (0.08)	1.48 (0.48)	1.26 (0.71)	2.00 (0.29)

**Table 5:** Matrix composition of the tested composites, which contained 45 wt% of silanized silica.

Material Signature	Base Material	Modification
Control	UDMA 40 wt% bis-EMA 40 wt% TEGDMA 20 wt%	None
CHMDI(2.5)_IPDI(2.5)		DEGMMA/CHMDI 2.5 wt% and DEGMMA/IPDI 2.5 wt%
CHMDI(5)		DEGMMA/CHMDI 5 wt%
CHMDI(10)		DEGMMA/CHMDI 10 wt%
IPDI(5)		DEGMMA/IPDI 5 wt%
IPDI(10)		DEGMMA/IPDI 10 wt%
CHINOX(0.5)		CHINOX SA-1 0.5 wt%
CHINOX(1.5)		CHINOX SA-1 1.5 wt%

UDMA—urethane dimethacrylate, bis-EMA—bisphenol A ethoxylateddimethacrylate, TEGDMA—triethyleneglycol dimethacrylate, CHINOX SA-1—bis(2,6-diisopropylphenyl)carbodiimide, DEGMMA/CHMDI—diethylene glycol monomethacrylate/4,4′-methylenebis(cyclohexyl isocyanate), DEGMMA/IPDI—diethylene glycol monomethacrylate/isophorone diisocyanate.

**Table 6:** Description of selected aging protocols used to evaluate the tested materials.

Ageing Protocol Signature	Description
control	24 h, 37 °C, distilled water
thermo_NaOH	7500 cycles, 5 °C and 55 °C, water and 7 days, 60 °C, 0.1 M NaOH
water_NaOH	5 days, 55 °C, water and 7 days, 60 °C, 0.1 M NaOH

## Data Availability

Data are available in a publicly accessible repository: Zenodo at: 10.5281/zenodo.7656550.

## Abbreviations

List of abbreviations:

bis-EMA	bisphenol A ethoxylateddimethacrylate,
CHINOX SA-1	bis(2,6-diisopropylphenyl)carbodiimide,
CHINOX(0.5)	addition of 0.5 wt% CHINOX SA-1,
CHINOX(1.5)	addition of 1.5 wt% CHINOX SA-1,
CHMDI(10)	addition of 10 wt% DEGMMA/CHMDI,
CHMDI(2.5)_IPDI(2.5)	addition of 2.5 wt% DEGMMA/CHMDI and 2.5 wt% DEGMMA/IPDI,
CHMDI(5)	addition of 5 wt% DEGMMA/CHMDI,
Control	24 h, 37 °C, distilled water,
DEGMMA/CHMDI	diethylene glycol monomethacrylate/4,4′-methylenebis(cyclohexyl isocyanate),
DEGMMA/IPDI	diethylene glycol monomethacrylate/isophorone diisocyanate
DTS	diametral tensile strength [MPa],
FS	flexural strength [MPa],
HV	hardness [-],
E	flexural modulus [GPa],
WS	water sorption [µg/mm^3^],
DC	degree of conversion [%],
IPDI(10)	addition of 10 wt% DEGMMA/IPDI,
IPDI(5)	addition of 5 wt% DEGMMA/IPDI,
MW	molecular weight [g/mol],
TEGDMA	triethyleneglycol dimethacrylate,
thermo_NaOH	aging for 7500 cycles, 5 °C and 55 °C, water and 7 days, 60 °C, 0.1 M NaOH,
UDMA	urethane dimethacrylate,
water_NaOH	aging for 5 days, 55 °C, water and 7 days, 60 °C, 0.1 M NaOH.
